# Riluzole and Prognostic Factors in Amyotrophic Lateral Sclerosis Long-term and Short-term Survival: A Population-Based Study of 1149 Cases in Taiwan

**DOI:** 10.2188/jea.JE20120119

**Published:** 2013-01-05

**Authors:** Charles Tzu-Chi Lee, Yi-Wen Chiu, Kai-Chen Wang, Chi-Shin Hwang, Kuan-Hsiang Lin, I-Ta Lee, Ching-Piao Tsai

**Affiliations:** 1 Department of Public Health, Kaohsiung Medical University, Kaohsiung, Taiwan; 2 Renal Care Faculty, Medicine College, Kaohsiung Medical University, Kaohsiung, Taiwan; 3 Nephrology Division, Internal Medicine Department, Kaohsiung Medical University Hospital, Kaohsiung, Taiwan; 4 Department of Neurology, Cheng Hsin General Hospital, Taipei, Taiwan; 5 Department of Neurology, Taipei City Hospital, Zhong-Hsiao Branch, Taipei, Taiwan; 6 Neurological Institute, Taipei Veterans General Hospital, Taipei, Taiwan; 7 Department of Physiology and Pharmacology, Chang Gung University, Kwei-San, Tao-Yuan, Taiwan

**Keywords:** amyotrophic lateral sclerosis, survival, riluzole, tracheotomy

## Abstract

**Background:**

Amyotrophic lateral sclerosis (ALS) is a rare disease in Taiwan; thus, estimation of ALS mortality is difficult. We evaluated factors associated with ALS survival in Taiwan.

**Methods:**

The study enrolled 1149 Taiwanese with a primary diagnosis of ALS during 1999–2008. Follow-up information was available for all patients; mean (SD) duration of follow-up was 2.91 (2.62) years. Medical interventions, including noninvasive positive pressure ventilation (NIPPV), tracheotomy, gastrostomy, and riluzole, were included in time-dependent survival analysis.

**Results:**

Of the 1149 ALS patients, 438 (38.12%) died during follow-up. Mortality in the first year was 16%, which was 13 times (95% CI 11.1–15.2) the age- and sex-standardized rate of the general population in Taiwan. The average annual crude mortality rate was 13.1% (person-years). Factors significantly associated with increased mortality were male sex, advanced age, rural residence, lower economic status, no tracheotomy, and no riluzole treatment. Significant predictors of long-term versus average survival were younger age at diagnosis, being a dependent or receiving social welfare, and NIPPV support. Significant predictors of short-term versus average survival were older age, being employed, no tracheotomy, and no riluzole use.

**Conclusions:**

The results support the use of riluzole to improve ALS survival. Patients who received riluzole and underwent tracheotomy had the best survival.

## INTRODUCTION

Amyotrophic lateral sclerosis (ALS)—a motor neuron disease—is a fatal neurodegenerative disorder. Without mechanical ventilation, death from respiratory failure usually occurs within 2 to 5 years after symptom onset.^1^ An increase in the prevalence and incidence of ALS has been observed in Hong Kong,^2^ Japan,^3^ Sweden,^4^ and possibly worldwide. Mechanical ventilation is effective in relieving chronic hypoventilation and prolonging life in ALS patients.^5^ The only medication used for treatment, riluzole, might improve quality of life and survival of ALS patients.^6^^–^^8^ However, the survival benefit of treatment comprising riluzole and tracheotomy with mechanical ventilation has not been investigated in Taiwan. Therefore, we investigated factors associated with survival of ALS patients in Taiwan.

## METHODS

### National health insurance in Taiwan

In 1995, the National Health Insurance (NHI) program, a government-run single-payer insurance system, was established in Taiwan. By December 2010, there were 23.074 million individuals enrolled in the program nationwide, with a coverage rate of 99.6%. The NHI bureau requires registration of all cases of serious disabling diseases (SDDs), such as chronic renal failure, myasthenia gravis, cancer, and ALS, before SDD certification can be granted. A group of neurology specialists at the Taiwan Bureau of National Health Insurance (BNHI) used the El Escorial criteria to review the medical records of ALS patients^9^ and found that 37 099 medical doctors and 553 neurology specialists were registered in 2008 in Taiwan. In addition, there were 790 621 individuals with SDD certificates in 2008, which constituted 3.4% of the total population.

### Sample

This study was a population-based retrospective cohort study that analyzed information from the National Health Insurance Research Database (NHIRD), which includes data on outpatient, ambulatory, inpatient, dental services and prescription drugs. ALS cases were identified by using code 335.20 of the International Classification of Disease, Ninth Revision (ICD-9). The study data comprised information from all medical claims by ALS patients during the period from January 1, 1999 to December 31, 2008. Only ALS patients with SDD certification were included. Patients with SDD certificates are eligible for exemption from insurance premiums and copayments. The approval of SDD certificates requires strict evaluation by the Department of Health, Executive Yuan, in Taiwan. In this study, all ALS cases were verified by linking encrypted identification numbers with SDD certificates. All ALS cases had SDD certificates and were followed until December 31, 2008, using the national mortality database.

### Statistical analyses

We used life table analysis to estimate mortality rates for different years during follow-up. The total mortality rate by year in the life table was calculated in relation to the number at risk, which is the number of survivors that entered the study interval minus half the number of patients that were lost or censored. Age- and sex-specific mortality rates from Taiwanese mortality statistics for the period from January 1, 1999 to December 31, 2008 were applied to the study sample to calculate expected number of deaths. The standardized mortality ratio (SMR) during the first 1 year of follow-up after ALS diagnosis was calculated as the ratio of observed to expected number of deaths. The crude mortality rate in the overall follow-up period was calculated by the person-years at risk method.

All cases were followed until December 31, 2008. Time-dependent Cox regression models were used to estimate hazard rates of ALS mortality according to sex, age, residence, insurance premium, geographic region, noninvasive positive pressure ventilation (NIPPV), tracheotomy, gastrostomy, and riluzole use (daily dose, according to World Health Organization recommendations, 100 mg). Residence was categorized as rural or urban. The insurance premium served as an indicator of economic status and was classified into 2 categories, namely, fixed premium or dependent and fixed income from salary per month. The fixed premium included patients requiring social welfare support, eg, low-income citizens and veterans. The dependent insurance group includes family members without a fixed income from salary. In addition, riluzole use was defined by daily dose and was the main time-dependent covariate. In regression analysis of time-dependent survival, the survival period for each individual was divided by a sequence of shorter survival intervals, each characterized by an entry and exit time.^10^ The date of the riluzole prescription was a time point that represented entry and exit times. For each survival period, survival time and daily riluzole dose were calculated. The period of time from ALS diagnosis to tracheotomy was also included in the analysis. The results for each risk factor are shown in unadjusted and adjusted analyses. The adjusted analysis included the following variables in the survival regression model: sex, age at ALS diagnosis, residence, insurance premium, geographic region, NIPPV, tracheotomy, gastrostomy, and riluzole use.

To test prognostic factors for long-term and short-term ALS survival, we analyzed only nonsurvivors, which were subdivided into those with long, average, and short survival, as in Zoccolella et al.^11^ All 438 nonsurvivors were classified in this manner. There were 111 long-term survivors (25% with longest survival), 111 short-term survivors (25% with shortest survival), and 216 average survivors. To identify predictors in 2 patient groups (long vs average survival and short vs average survival) and compare our data with those of recent studies, we used logistic regression to identify prognostic factors for long- and short-term ALS survival. The adjusted variables were the same as those included in the previous survival regression analysis. Analyses were carried out using SAS version 9.2.

## RESULTS

### Sample characteristics

A total of 1149 ALS patients received SDD certification between January 1, 1999 and December 31, 2008. Follow-up information was available for all patients, and mean (SD) duration of follow-up was 2.91 (2.62) years. No patient withdrew at the baseline, and the sample consisted of 715 (62.23%) men and 434 (37.77%) women. Mean (SD) age at diagnosis was 56.27 (14.15) years. Nearly 78% of the study sample lived in urban areas of Taiwan, and around 53% lived close to Taipei city in northern Taiwan. In addition, almost 59% of the study sample had no regular income from salary. Furthermore, during the study period, 193 patients (16.8%) received NIPPV and 241 (20.97%) underwent tracheotomy, and all patients who underwent tracheotomy required mechanical ventilation. Only 44 patients (3.8%) underwent gastrostomy. There were 698 riluzole users (60.75%) during the study period (Table 1).

**Table 1.** Characteristics of 1149 ALS patients in Taiwan, 1999–2008

Characteristic	*n*	%
Sex		
Female	434	37.77
Male	715	62.23
Age at ALS diagnosis, y		
0–39	128	11.14
40–49	236	20.54
50–59	284	24.72
60–69	310	26.98
70+	191	16.62
Residence		
Rural	254	22.11
Urban	895	77.89
Insurance premium		
Dependent or receiving social welfare	674	58.66
From salary	475	41.34
Geographic region		
North	602	52.39
Central	208	18.10
South	299	26.02
East	40	3.48
NIPPV		
No	956	83.20
Yes	193	16.80
Tracheotomy with mechanical ventilation		
No	908	79.03
Yes	241	20.97
Gastrostomy		
No	1105	96.17
Yes	44	3.83
Riluzole use		
No	451	39.25
Yes	698	60.75

NIPPV: Noninvasive positive pressure ventilation.

### Mortality rate after ALS diagnosis

**Figure 1.**
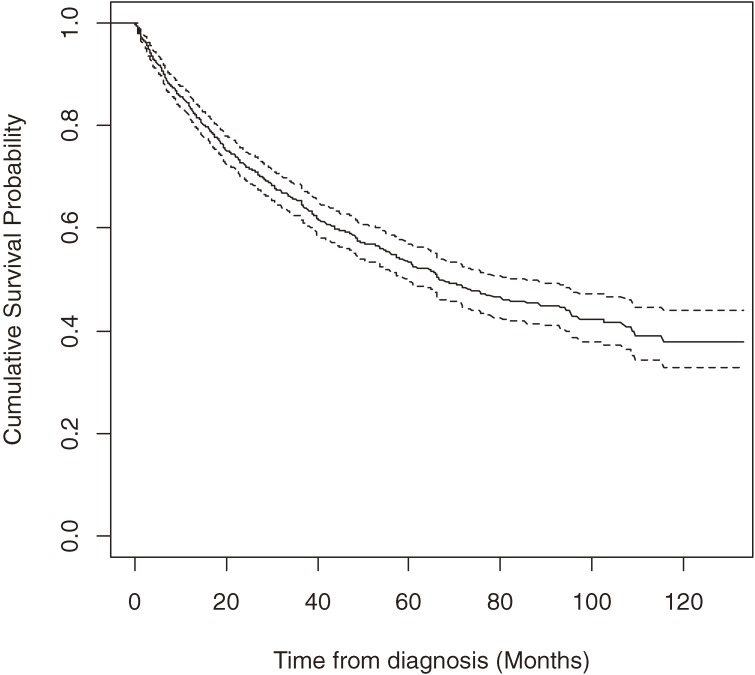
Kaplan-Meier survival curves from diagnosis for patients with amyotrophic lateral sclerosis in Taiwan, 1999–2008 (*n* = 1149). Solid line: predicted cumulative survival. Dashed line: 95% CI.

Of the 1149 ALS patients, 438 (38.12%) died during follow-up. Mean overall survival time was 67.75 months (95% CI 64.50–70.99), and median survival time was 66.6 months (95% CI 59.10–82.33) (Figure 1).

The mortality rate during the first year was 16%, which was 13 times (95% CI, 11.1–15.2) the age- and sex-standardized rate of the general population of Taiwan. Although the mortality rate continued to increase steadily, the amplitude was the highest during the first year (Table 2). The average annual crude mortality rate was 13.1% (person-years) during the overall follow-up period.

**Table 2.** Mortality risk by timing of death after ALS diagnosis (*n* = 1149)

Timing after ALS diagnosis, y	No. exposed	No. of deaths	Mortality risk (%)		

			Estimate	Lower limit for 95%CI	Upper limit for 95%CI
0–1	1079	173	16.0	13.8	18.2
1–2	768	105	13.7	11.2	16.1
2–3	543	52	9.6	7.1	12.0
3–4	399.5	43	10.8	7.7	13.8
4–5	291.5	26	8.9	5.6	12.2
5–6	212	19	9.0	5.1	12.8
6–7	145.5	8	5.5	1.8	9.2
7–8	101	6	5.9	1.3	10.5

### Factors associated with mortality after ALS diagnosis

The main findings of this study are shown in Table 3. The unadjusted Cox regression model showed that factors significantly associated with mortality included older age, rural residence, lower economic status, no tracheotomy, and no riluzole treatment. In the fully adjusted model, the final independent predictors were male sex, older age, rural residence, lower economic status, no tracheotomy, and no riluzole treatment. There were insufficient data on the survival difference between patients with and without a gastrostomy. In total, 193 ALS patients received NIPPV. However, there was insufficient evidence of a significant survival difference between patients with and without NIPPV. In the fully adjusted model, the mortality rate among patients receiving riluzole (100 mg) was reduced by almost 66% (HR = 0.34; 95% CI 0.24–0.49). Mean (SD) duration of riluzole use among the 698 users was 18.17 (23.24) months during the study period. ALS patients who underwent tracheotomy had a 48% reduction in mortality risk (HR = 0.52; 95% CI 0.36–0.77) in the fully adjusted model.

**Table 3.** Time-dependent Cox regression analysis^a^ of ALS mortality (*n* = 1149)

Variable	Unadjusted analysis		Adjusted analysis	
	
	Hazard ratio (95%CI)	*P* value	Hazard ratio (95%CI)	*P* value
Sex				
Female	1.00		1.00	
Male	1.19 (0.98–1.45)	0.077	1.27 (1.02–1.58)	0.026
Age, y	1.04 (1.03–1.05)	<0.001	1.04 (1.02–1.04)	<0.001
Residence				
Urban	1.00		1.00	
Rural	1.66 (1.35–2.05)	<0.001	1.43 (1.02–1.86)	0.007
Geographic region				
North	1.00		1.00	
Central	1.16 (0.90–1.49)	0.255	0.97 (0.72–1.3)	0.838
South	1.13 (0.90–1.41)	0.284	1.10 (0.86–1.42)	0.452
East	1.39 (0.86–2.26)	0.176	0.97 (0.61–1.54)	0.905
Insurance amount				
From salary	1.00		1.00	
Dependent or receiving social welfare	1.35 (1.15–1.55)	<0.001	1.26 (1.05–1.47)	<0.001
NIPPV				
No	1.00		1.00	
Yes	1.25 (0.94–1.66)	0.122	1.16 (0.87–1.45)	0.129
Tracheotomy with mechanical ventilation				
No	1.00		1.00	
Yes	0.62 (0.45–0.87)	0.005	0.52 (0.36–0.77)	0.001
Gastrostomy				
No	1.00		1.00	
Yes	0.68 (0.37–1.28)	0.236	0.80 (0.44–1.50)	0.501
Riluzole (per DDD^b^)	0.32 (0.22–0.45)	<0.001	0.34 (0.24–0.49)	<0.001

^a^Other confounders, such as symptoms and site of onset, were not included in the dataset and thus are not included in the table.

^b^DDD: World Health Organization defined daily dose, 100 mg. NIPPV: noninvasive positive pressure ventilation.

### Effect of riluzole and tracheotomy on survival

**Figure 2.**
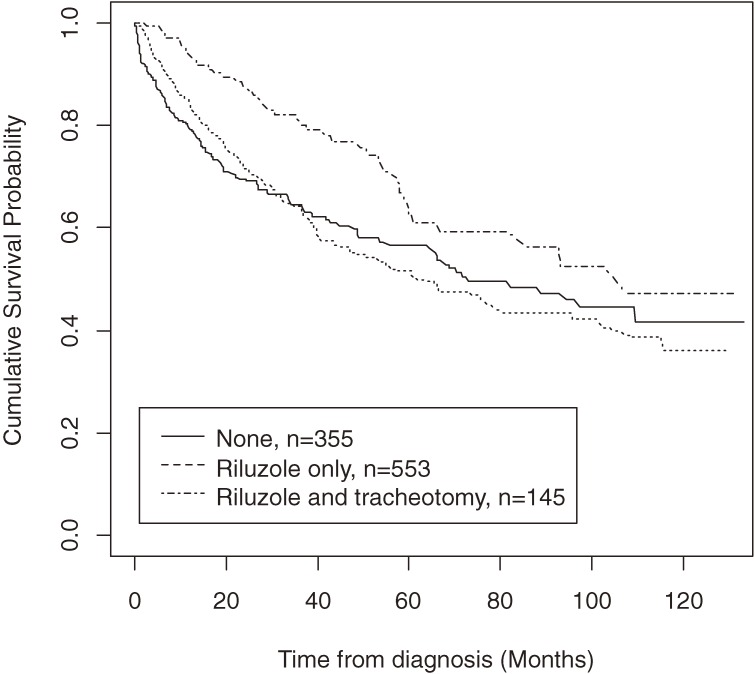
Kaplan-Meier survival curves from diagnosis for patients with amyotrophic lateral sclerosis treated with riluzole and tracheotomy plus mechanical ventilation in Taiwan, 1999–2008 (*n* = 1149).

The subjects were divided into 3 groups based on their riluzole and tracheotomy status: (1) none (no riluzole and no tracheotomy, *n* = 355), (2) riluzole only (*n* = 553), and (3) riluzole and tracheotomy (*n* = 145). In addition, 96 patients underwent tracheotomy without riluzole treatment. The combination of tracheotomy and riluzole resulted in better survival (log-rank test *P* = 0.027), and patients who received both had better survival than those who received neither (log-rank test *P* = 0.004). In addition, patients who received both also had better survival than those who received riluzole only (log-rank test *P* = 0.010). The combination of riluzole and tracheotomy resulted in the best survival (Figure 2).

### Predictors of long-term and short-term survival

Among the 438 nonsurvivors, mean (SD) survival time was 57.05 (19.45) months for the 111 long-term survivors (25% with longest survival). Because this analysis focused on the 438 nonsurvivors, mean survival time was shorter than the overall survival time of the 1149 ALS patients. Mean (SD) survival time for the 111 short-term survivors (25% with shortest survival) was 6.50 (1.9) months. The other 216 ALS patients were classified as average survivors, and their mean (SD) survival time was 17.66 (7.8) months. Significant predictors of long-term versus average survival were younger age at diagnosis, being a dependent or receiving social welfare, and NIPPV support (Table 4A ). Significant predictors of short-term versus average survival were older age, being employed, no tracheotomy, and no riluzole use (Table 4B).

**Table 4.** Multivariate logistic regression analysis of predictors of long- and short-term survival in Taiwanese ALS patients, 1999–2008

(A) Long-term vs average survival		

Predictors	Odds-ratio (95% CI)	*P* value
Age at ALS diagnosis, y	0.965 (0.946–0.984)	<0.001
Insurance premium		
From salary	1.000	
Dependent or receiving social welfare	1.970 (1.143–3.396)	0.015
Non-invasive positive pressure ventilation		
No	1.000	
Yes	2.033 (1.116–3.704)	0.020


(B) Short-term vs average survival		

Predictors	Odds ratio (95% CI)	*P* value
ALS diagnosis age, y	1.024 (1.004–1.045)	0.020
Insurance premium		
From salary	1.000	0.006
Dependent or receiving social welfare	0.495 (0.299–0.819)	
Tracheotomy with mechanical ventilation		
No	1.000	
Yes	0.361 (0.177–0.735)	0.005
Riluzole use		
No	1.000	
Yes	0.457 (0.279–0.748)	0.002

## DISCUSSION

This is the first report of survival time for ALS patients in Taiwan. Mean age at ALS diagnosis was 56.27 years, which was higher than that among Hispanics (47.5),^12^ Argentineans (55),^13^ and cohorts in India (46.2),^14^ but lower than in Japan (64.8).^15^ Mean/median survival time for ALS in Taiwan (67.8/66.6 months) was similar to that in Hispanics^12^ (mean = 68.6 months), higher than that in Italians (median = 16 months)^16^ and Irish (median = 16.4),^17^ and lower than that reported in Indians (median = 114.8 months).^14^ As in previous studies,^12^^,^^16^ men had poorer survival. However, in a recent review, sex had no clear effect on survival.^18^ In the present study, survival was shorter in men than in women. The reason for this sex difference is unclear.

The present findings suggest that riluzole increases survival among ALS patients.^7^ Riluzole is a standard therapy for ALS in Taiwan. Although it is reimbursed by the National Health Institute in Taiwan, about 40% of patients do not use riluzole because they think it will not cure ALS and/or because of its adverse effects. In addition, 298 (42.7%) of 698 patients in this study stopped using riluzole during follow-up. However, in our time-dependent survival analysis, we modeled the effect of riluzole dose on survival for different durations of administration, and the effect of riluzole cessation was addressed. In addition, because riluzole therapy is reimbursed and is recommended by the National Health Institute in Taiwan, physicians were unlikely to withhold riluzole treatment.

Riluzole is a neuroprotective drug that blocks glutamatergic neurotransmission in the central nervous system. It inhibits release of glutamic acid from cultured neurons, from brain slices, and from corticostriatal neurons in vivo. It is believed that these effects are partly due to inactivation of voltage-dependent sodium channels on glutamatergic nerve terminals, as well as to activation of a G-protein–dependent signal transduction process.^19^ A double-blind, placebo-controlled, multicenter study of 959 patients with clinically probable or definite ALS of less than 5 years' duration were randomly assigned to treatment with placebo or 50 mg, 100 mg, or 200 mg riluzole daily. Randomization was stratified by treatment center and site of disease onset (bulbar or limb). Efficacy and safety results suggested that the 100 mg dose of riluzole had the best benefit-to-risk ratio. Riluzole is well tolerated and increases survival of ALS patients.^20^

Previous research indicates that a tracheostomy improves quality of life for ALS patients, among whom it has been shown to be useful in predicting survival.^21^ In addition, tracheotomy was found to reduce mortality risk.^22^ In Italy, 134 ALS patients (10.6%) underwent tracheotomy^23^; however, only 6.7% of men and 3.8% of women underwent invasive procedures via tracheostomy in Norway and Sweden.^24^ In contrast, about 20% of Japanese ALS patients undergo tracheotomy.^25^ In our study, 21.0% of ALS patients underwent tracheotomy. It appears that tracheostomy use is higher in Asian than in European countries, which might be due to ethnic and cultural differences.

In an Italian population-based study, Zoccolella et al reported that younger age, longer interval from symptom onset to diagnosis, and clinical predominance of upper motor signs predicted long-term survival.^11^ In the present study, multivariate logistic regression analysis showed that significant predictors of long-term and average survival were younger age at diagnosis, being a dependent or receiving support from social welfare, and NIPPV support. Although there was insufficient evidence that NIPPV support improved survival in an analysis of all 1149 ALS cases, NIPPV was a predictor of long-term survival in this study. Although riluzole therapy plus tracheotomy resulted in the longest survival, the interaction of tracheotomy and riluzole use with survival requires further investigation. The association of lower socioeconomic status and rural residence with relatively poor survival showed that social welfare support of these patients is still very important in Taiwan.

This was an observational population-based study. Thus, we recommend a randomized clinical trial in Taiwan to confirm our findings. The limitations of this study include the absence of some important predictors, such as ALS symptoms. Disease onset and diagnosis may differ according to the economic status and residence of patients, as these variables could affect access to neurologists. Unfortunately, date of ALS onset was not available in the dataset. In addition, we were unable to distinguish familial and primary ALS.
